# A Phytocomplex Consisting of *Tropaeolum majus* L. and *Salvia officinalis* L. Extracts Alleviates the Inflammatory Response of Dermal Fibroblasts to Bacterial Lipopolysaccharides

**DOI:** 10.1155/2020/8516153

**Published:** 2020-05-21

**Authors:** Tunde Jurca, Ioana Baldea, Gabriela Adriana Filip, Diana Olteanu, Simona Clichici, Annamaria Pallag, Laura Vicaş, Eleonora Marian, Otilia Micle, Carmen Bianca Crivii, Mariana Mureşan

**Affiliations:** ^1^Department of Pharmacy, Faculty of Medicine and Pharmacy, University of Oradea, 29 Nicolae Jiga Street, 410028 Oradea, Romania; ^2^Department of Physiology, “Iuliu Hatieganu” University of Medicine and Pharmacy, 1-3 Clinicilor Street, 400006 Cluj-Napoca, Romania; ^3^Department of Preclinical Disciplines, Faculty of Medicine and Pharmacy, University of Oradea, 10 Piata 1 Decembrie Street, 410073 Oradea, Romania; ^4^Morphology Department, “Iuliu Hatieganu” University of Medicine and Pharmacy, 3-5 Clinicilor Street, 400006 Cluj-Napoca, Romania

## Abstract

**Background:**

The antimicrobial activity and effects of a phytocomplex consisting of *Tropaeolum flos* (T) and *Salviae folium* (S) extracts on the cytokine levels and transcription factors on dermal fibroblast BJ exposed to bacterial lipopolysaccharides were examined.

**Methods:**

In order to select the most optimal combination ratio of the two extracts for using *in vitro*, the physicochemical characterization of vegetal extract mixtures was performed and the antioxidant and antibacterial activities were evaluated on five different formulations of T : S, namely, 1 : 1, 1 : 2, 2 : 1, 3 : 1, and 1 : 3. The best combination of bioactive compounds with regard to antioxidant and antibacterial activities (T : S 1 : 2) was selected for *in vitro* evaluation of the anti-inflammatory effect. Human dermal fibroblast BJ cells were treated with two doses of the extract mixture and then exposed to bacterial lipopolysaccharides (LPS). The levels of the cytokines involved in inflammatory response, namely, interleukin- (IL-) 6, tumor necrosis factor- (TNF-) *α*, IL-31, and IL-33, were quantified by ELISA, and the expression of transcription factors, namely, signal transducer and activator of transcription (STAT) 3, nuclear factor kappa B (NF*κ*B), and phosphorylated NF*κ*B (pNF*κ*B), were evaluated by western blot analysis.

**Results:**

The results have shown that the mixture of T : S 1 : 2 exhibited significant antibacterial effects on *Staphylococcus aureus* ATCC 25923. LPS exposure increased the cytokine levels in BJ cells and enhanced the NF*κ*B expression. The pretreatment of BF cells exposed to LPS with the two doses of the extract mixture markedly inhibited the increase of IL-33 and TNF-*α* levels and amplified the NF*κ*B expression and its activation, especially with the high dose. The low doses of the extract reduced NF*κ*B expression but increased its activation.

**Conclusions:**

These experimental findings suggest that the mixture of T : S 1 : 2 can exert some protection against bacterial infections and inflammation induced by LPS in BJ cells being a good therapeutic option in related conditions associated with inflammation.

## 1. Introduction

In the recent years, more and more studies focused on the pathogenetic mechanisms of atopic dermatitis (AD) and its treatment. Atopic dermatitis is one of the most common chronic inflammatory skin diseases whose incidence has increased considerably over the past decades, especially in industrialized countries. Thus, the prevalence of atopic dermatitis varies between 7% and 30% in children and between 1% and 10% in adults and evolves with a significant decrease in the quality of their life [1, 2].

AD is a complex condition with multifactorial aetiology, characterized by periods of exacerbation and remission with dry skin (xerosis), pruritus, and increased loss of transepidermal water [3]. With all the remarkable progress made, the cause of AD has not been completely elucidated. Most studies consider that the disease appears as a result of the combined action of genetic features, barrier dysfunction, and environmental, immunological, and biochemical factors. The key factor in the pathogenesis of this disease is the alteration of the skin barrier due to loss of the functions of filaggrin, a structural protein, important for cornification and skin hydration [4]. In addition, the affected skin is deficient in ceramide and has increased levels of endogenous proteolytic enzymes, responsible for transepidermic loss of water and alteration of cutaneous barrier function [5].

Several studies have shown the involvement of the imbalance of Th2 to Th1 cytokines and consequently the increased systemic response of Th2-type lymphocytes which initiate and maintain skin inflammation [6] and also enhance the hyperreactivity to environmental factors. Dysfunction of Th2 lymphocytes and cytokines released by them, namely, IL-4, IL-5, and IL-13, lead to increased immunoglobulin (Ig) E production, which amplifies the local inflammation and deteriorates the skin barrier function. In addition, Th17 and Th22 lymphocytes released the IL-17, IL-19, and IL-22 cytokines known to be involved in the pathogenesis of atopic dermatitis [7, 8]. As a response to changes in the skin barrier function, the keratinocytes secreted cytokines, such as stromal lymphopoietin, IL-25, and IL-33, which in turn activate Th2 lymphocytes and congenital lymphoid cells (ILC2) [9].

Several papers have suggested that new tissue-derived cytokines such as IL-33 have a significant role in AD [10, 11]. IL-33 belongs to the IL-1 family, and it is secreted by damaged tissues and activates Th2 lymphocytes, mast cells, basophils, and eosinophils to produce Th2-type cytokines [12, 13]. It seems that IL-33 would have a dual function: one by extracellular action, as a member of the IL-1 cytokine family, and the other by intracellular action, as nuclear factor that regulates gene expression [14–17].

Several evidences have shown an association of IL-31, a cytokine secreted by Th2 cells, mast cells, macrophages, and dendritic cells, with severe pruritus in inflammatory diseases including AD. The blocking of its expression mitigates the scratching behaviour in AD and chronic spontaneous urticaria [18, 19]. The expression of IL-31RA in human keratinocytes and macrophages is low in an unstimulated condition [21], and it is upregulated by interferon *γ* and toll-like receptor 2/toll-like receptor 1 agonists [20]. Binding of IL-31 to its receptor determines the phosphorylation and activation of the mitogen-activated protein kinase (MAPK), c-Jun N-terminal kinase (JNK), and STAT pathways [22].

In recent years, there has been a growing demand for medicinal and aromatic herbs in the world due to the increased content in biologically active substances frequently used in the pharmaceutical, cosmetic, and food industries. Considerable research pointed out the potential effectiveness of natural compounds in biology and medicine due to their potent antioxidant, anti-inflammatory, and immunomodulatory properties [23–25]. These can be benefited by diet or through skin application, diminishing symptoms and inhibiting the development of various skin diseases without having the side effects of corticosteroids. *Salvia officinalis* L. (common sage) and *Tropaeolum majus* L. (garden nasturtium) are two of the most promising species for medical applications. *Salvia officinalis* L., the common sage or garden sage, is a perennial herb of the Lamiaceae family. Several studies demonstrated that *Salvia officinalis* L. had different biological activities which have antibacterial, virustatic, fungistatic, astringent, and antihydrotic effects [26–30]. The effects were due to the triterpenes oleanolic and ursolic acids and diterpene carnosol from a composition with anti-inflammatory properties and antiprotease and antimetastatic activities [31, 32]. In other *Salvia* species such as the *S. verticillata* subsp. *amasica* extract, the main compound identified was rosmarinic acid which explained the strong antioxidant activity [33].

*Tropaeolum majus* L., a garden nasturtium of the Tropaeolaceae family, is an annual plant originally from the Andes, Bolivia, Peru, and Colombia, known for the properties of the aerial part of *Tropaeoli herba* or the flowers of *Tropaeoli flos* [3, 34]. The *Tropaeolum majus* L. extract demonstrated antioxidant and anti-inflammatory activities due to its content of polyphenols, flavonoids, and ascorbic acid in different experimental models [35, 36].

Based on these data, the study is aimed at evaluating the physicochemical properties of the mixture of the two vegetal extracts obtained from crops harvested in Bihor County, Romania's northern area, as well as evaluating the *in vitro* antioxidant capacity in order to assess their biological activities on human dermal fibroblast BJ exposed to bacterial lipopolysaccharides (LPS). It is known that LPS is a major component of the outer membrane of gram negative bacteria and a potent inductor of inflammation. Moreover, its bioactive moiety endotoxin can be measured in dust collected from homes. In addition, there are some papers which demonstrated a positive relationship between exposure to endotoxin and the high incidence of atopic dermatitis [37]. Therefore, we used BJ cells exposed to LPS as a model to simulate *in vitro* atopic dermatitis.

We also tested the antimicrobial activity against different bacteria using the reference microbial strains and also the human clinical isolates from patients with infections or from the hospital environment. The evaluation of antimicrobial activity of the mixture of two extracts was important due to the increased incidence of infections in atopic dermatitis and the role played by infection as a trigger for disease exacerbation.

## 2. Materials and Methods

### 2.1. Reagents

Galic acid, quercetin, 2,2-diphenyl-1-picryl-hydrazyl (DPPH free radical), 2,2′-azino-bis (3-ethylbenzothiazoline-6-sulfonate) (ABTS), the ferric-reducing antioxidant power (FRAP), Folin Ciocâlteu reagents, and lipopolysaccharides from *Escherichia coli* O111:B4 were purchased from Sigma-Aldrich Chemicals GmbH (Germany). The Bradford reagent was from Merck KGaA (Darmstadt, Germany). ELISA tests for the evaluation of IL-6, IL-31, IL-33, TNF-*α*, and STAT3 were from R&D Systems Inc. (Minneapolis, MN, USA). The goat polyclonal IgG antibody for NF*κ*B and pNF*κ*B and the secondary antibody mouse anti-goat and glyceraldehyde 3-phosphate dehydrogenase (GAPDH) were bought from Santa Cruz Biotechnology Inc. (Santa Cruz, CA, USA). All the chemicals used were of analytical grade.

### 2.2. Plant Materials and Preparation of the Extract

*Salvia officinalis* L. and *Tropaeolum majus* L. plants were harvested from crops in Bihor County, Romania, in June–July 2018. The voucher specimens from both species were deposited in the Herbarium Pharmacy Department, University of Oradea (registered in the NYBG William and Lynda Steere Herbarium, code: UOP 05212 for *Salvia officinalis* L. and code: UOP 05073 for *Tropaeolum majus* L.). The plant material was dried after harvesting using an FDK 24 DW Gorenje dryer at 40°C for total moisture removal. The next step further, which was the vegetable product shredding, is one of the important factors in achieving the extractive preparations. By increasing the contact surface of the plant and solvent product, the extraction time is reduced and the extraction efficiency is increased. The degree of grinding of the plant products was chosen according to the European Pharmacopoeia (Ph Eur. 9th) and the Romanian Pharmacopoeia (FR X) and was passed through sieve I after a preliminary grinding with an electrical mill [38, 39].

The extracts were obtained by maceration at room temperature according to the European Pharmacopoeia (Ph. Eur. 9th) and Romanian Pharmacopoeia (FR X). The method was applied to both extracts of the flowers of *Tropaeolum majus* L. (denoted T) and extracts of the leaves of *Salvia officinalis* L. (denoted S). Each plant product was subjected to 30^c^ ethyl alcohol maceration at room temperature (20°C) for 24 hours; the plant/30^c^ alcohol ratio was 20% (*m*/*m*). After completion of the extraction, they were decanted and filtered using nylon filter paper, 100 *μ*m.

### 2.3. Physicochemical Properties of the Mixture of Extracts

In previous papers, we performed the evaluation of the polyphenol and flavonoid content of each extract by reversed-phase HPLC (RP-HPLC) [35, 40]. The RP-HPLC analysis of the *Tropaeolum majus* L. extract has shown the following composition: in phenolic acids—gallic, caffeic, syringic, synapic, vanillic, p-coumaric, and ferulic; and in flavonoids—catechin hydrate, rutin trihydrate, naringenin, luteolin, quercetin dihydrate, epicatechin, and myricetin [41]. The *Salvia officinalis* L. extract has an increased content of gallic acid, epicatechin, rutin, p-coumaric acid, luteolin, and quercetin.

Polyphenolic compounds and flavonoids have beneficial properties, acting as antioxidants in a biological system under oxidative stress conditions. In order to select the optimal combination ratio of the two extracts, several T : S combinations were used, namely, 1 : 1, 1 : 2, 2 : 1, 3 : 1, and 1 : 3. For this study, we evaluated the content of bioactive compounds based on their antioxidant properties demonstrated by *in vitro* methods.

#### 2.3.1. Determination of the Content in Polyphenolic Compounds

Determination of the content in polyphenolic compounds was performed by the Folin-Ciocâlteu method, the results being expressed in gallic acid (GAE) equivalents (mg/mL). In order to achieve this determination, 100 *μ*L of fluid extract was taken and mixed with 1750 *μ*L distilled water, 200 *μ*L of Folin-Ciocâlteu reagent (diluted 1 : 10 *v*/*v*), and 1000 *μ*L of 15% Na_2_CO_3_ solution and then kept at room temperature, away from light, for two hours. Then, the absorbance was measured at a wavelength of 765 nm using a UV-Vis spectrophotometer. The calibration curve was linear for the concentration range of 0.1-0.5 mg/mL for gallic acid. The content of the total polyphenols in the extracts is expressed as mg equivalent of gallic acid (GAE)/g dry weight extract (DW) [35].

#### 2.3.2. Determination of Total Flavonoids

Determination of total flavonoids was performed by the colourimetric method [35]. On this line, 1 mL of sample was taken and mixed with 4 mL of distilled water and placed in a 10 mL volumetric flask. Then, 3 mL of 5% NaNO_2_ solution was added, and after 5 minutes, 0.3 mL of 10% AlCl_3_ solution was added. After a further 6 minutes, 2 mL of 1 M NaOH was added. The flask was quenched to the mark with distilled water, and the absorbance was read at 510 nm. The calibration curve was used using the quercetin standard. The equation of the calibration curve is *y* = 56.818571*x* − 0.066498 (*R*^2^ = 0.9983), where *x* represents the absorbance and *y* represents mg quercetin.

### 2.4. Evaluation of the Antioxidant Activity of the Mixture of Extracts

A number of analytical methods have been developed to determine the antioxidant activity of natural products that are generally based on the reaction between an antioxidant species and a chromogenic compound. The antioxidant capacity of the extracts was evaluated by the following methods: DPPH (2,2-diphenyl-1-picryl-hydrazyl), ABTS (2,2′-azinobis (3-ethylbenzothiazoline-6-sulfonic acid), and ferric-reducing antioxidant power (FRAP).

#### 2.4.1. DPPH Method

The DPPH method is a spectrophotometric method widely used to test the ability of compounds to remove free radicals or their ability to donate hydrogen. The activity of capturing 2,2-diphenyl-1-picryl-hydrazyl radicals (DPPH) was determined using the method proposed by Pallag et al. [41]. Thus, at a volume of 100 mL of plant extract, 2900 mL of DPPH methanolic solution (80 mM) was added. The absorbance of the resulting solution was read at 515 nm after 5 minutes. The following equation was used to determine the DPPH inhibitory capacity (%):

(1) $$\begin{array}{c} \text{DPPH inhibition }\left(\%\right)=\frac{\left[A_{\text{o}}-A_{\text{s}}\right]\times \left.100\right]}{A_{\text{o}}}, \end{array}$$

where *A*_o_ is absorption blank and *A*_s_ is sample absorbance at 515 nm.

#### 2.4.2. ABTS Method

The ABTS method has been widely used to evaluate antioxidant activity and uses the method of Arnao et al. [42]. Briefly, ABTS^+^ was produced by reacting the ABTS solution (7 mM) with 2.45 mM potassium persulphate, keeping the mixture in the dark at room temperature for 16 hours. The stock solution of ABTS was diluted to obtain an absorbance of 0.70 ± 0.02 at 734 nm. After the addition of 25 *μ*L extract in 2.5 mL of diluted ABTS^+^, the mixture was homogenized very well (using Vortex) for 30 seconds and the interaction between antioxidants and ABTS^•+^ was determined spectrophotometrically at 734 nm, exactly 1 minute after homogenization. Trolox was used and a standard linear curve was obtained between 0.125 and 2 mmol/L Trolox. The ABTS value was obtained using the calibration curve equation: *y* = 1629*x* + 98.94 (*R*^2^ = 0.998), where *x* is absorbance and *y* is mmol Trolox equivalents.

#### 2.4.3. Ferric-Reducing Antioxidant Power (FRAP) Method

The ferric-reducing antioxidant power (FRAP) method is a simple spectrophotometric method that tests the antioxidant potency of the samples taken in the study and is based on the reduction of the ferric tripyridyl-triazine complex to the ferrous tripyridyl triazine complex (Fe (III)-TPTZ) by a pH-reducing agent [40]. Stock solutions include 300 mM acetate buffer; 270 mg of FeCl_3_·6H_2_O dissolved in 50 mL of distilled water; 150 mg of TPTZ; and 150 mL of HCl, dissolved in 50 mL of distilled water. The FRAP solution obtained was freshly prepared by mixing 50 mL of acetate buffer solution, 5 mL of FeCl_3_·6H_2_O solution, and 5 mL of TPTZ solution. The vegetable extracts (100 mL) were allowed to react with 500 mL of FRAP solution and 2 mL of distilled water for 1 hour, away from light. The final coloured product (ferric tripyridyl-triazine complex) was quantitated by absorption into the VIS at 595 nm. Trolox was used as the antioxidant positive control, and a standard linear curve of between 50 and 500 mmol/L of Trolox was obtained. The FRAP value was obtained using the equation based on the following calibration curve: *y* = 0.0157*x* + 0.0549 (*R*^2^ = 0.9981), where *x* is absorbance and *y* is mmol of Trolox equivalents.

### 2.5. Antimicrobial Activity

*In vitro* testing of the antimicrobial activity of the mixture of two plant extracts on bacteria was done by the diffusion method of Kirby-Bauer [43]. We used two reference strains from the American Type Culture Collection (ATCC), namely, *Staphylococcus aureus* ATCC 25923 and *Streptococcus pneumoniae* ATCC 49619 and two wild strains, namely, *Streptococcus pyogenes* and *Streptococcus agalactiae*—isolated from human clinical cases. Culture media (Mueller-Hinton (Oxoid) for *Staphylococci* and Mueller-Hinton Agar 2 + 5% sheep blood (BioMérieux) for *Streptococci*) were inoculated with standardized bacterial inoculum (0.5 McFarland units). After 10-15 minutes, 6 sterile filter papers (HiMedia Laboratories, Ref. SD067-5CT) 6 mm in diameter were placed on each Petri plate and 20 microliters of plant extract were impregnated into each disk. For each extract, the samples were worked in triplicate to minimize errors. We utilized standard penicillin disks (10 U, Oxoid) as a positive control, and paper disks smeared with distilled water (20 *μ*L) as a negative control. After incubating the media at 37°C for 18 hours, the diameters of the inhibition zones were measured with a ruler and the arithmetic mean for each extract was calculated.

### 2.6. Cell Cultures

The assays were performed on normal human dermal fibroblast BJ (ATCC, Gaithersburg, Maryland USA). Cell culture medium was DMEM (Dulbecco's modified Eagle's medium), supplemented with 5% FBS (foetal bovine serum), antibiotics, and antimycotics; all reagents were purchased from Sigma-Aldrich Chemicals GmbH (Heidelberg, Germany).

#### 2.6.1. Viability Assay

Cell survival was assessed through the colourimetric measurement of formazan, a coloured compound synthesized by viable cells, using the CellTiter 96® AQueous Nonradioactive Cell Proliferation Assay (Promega Corporation, Madison, USA). The dermal fibroblast BJ cultures were cultivated at a density of 10^4^/wells in 96-well plaques (TPP, Trasadingen, Switzerland) for 24 h, then exposed to the plant extracts in five different formulations, according to the following ratios between *Tropaeolum majus* (T) and *Salvia officinalis* extracts (S), respectively: (1) 1 : 1, (2) 1 : 2, (3) 2 : 1, (4) 3 : 1, and (5) 1 : 3. Concentrations ranging between 0 and -500 *μ*g GAE/mL of polyphenols were prepared from each extract in medium immediately before use. The LPS impact on viability was also tested, similarly to the extracts by exposing BJ cells to different LPS concentrations, between 0 and 10 *μ*g/mL. Cells were either exposed only to the extracts for 24 h, or, following the extracts' exposure, cells were washed and further exposed to LPS (lipopolysaccharides from *Escherichia coli*) in a concentration of 10 *μ*g/mL for an additional 24 h, then viability was measured colourimetrically, using an ELISA plate reader (Tecan, Männedorf, Switzerland) at 540 nm. The dose of 10 *μ*g/mL for LPS was chosen based on the doses used in the literature including ex vivo models [44]. All the experiments were done in triplicate. Untreated cell cultures were used as controls. Results are presented as OD540.

#### 2.6.2. Cell Lysates

The BJ cells, seeded on Petri dishes at a density of 10^4^/cm^2^, were exposed for 24 h to T : S 1 : 2 extract in concentration of 0.1 *μ*g polyphenols/mL (*D*_1_) and 0.01 *μ*g polyphenols/mL (*D*_2_) respectively, and then to LPS 10 *μ*g/mL for an additional 24 h, or only extract or LPS. Untreated cells were used as controls. Cells were washed following exposure, and afterwards, lysates were prepared as previously described [45]. Protein concentrations were determined by the Bradford method according to the manufacturer's specifications (Bio-Rad, Hercules, CA, USA), using albumin bovine serum as standard. For all assays, the lysates were corrected by total protein concentration.

#### 2.6.3. Inflammation Marker Assessment

Inflammation was assessed by the measurement of IL-31, IL-33, IL-6, TNF-*α*, and STAT3 levels using ELISA immunoassay kits from R&D Systems Inc. (Minneapolis, MN, USA). In addition, the expressions of transcription factor NF*κ*B and its phosphorylated form (pNF*κ*B) were evaluated by western blot analysis. For western blot, a 20 *μ*g protein/lane was separated by electrophoresis on SDS-PAGE gels and then transferred to polyvinylidene difluoride membranes (Bio-Rad Mini-PROTEAN System from Bio-Rad) as previously described [46]. Blots were blocked and then incubated with antibodies against NF*κ*B, pNF*κ*B p65 (Ser536) (93H1), and GAPDH and then further washed and incubated with corresponding secondary peroxidase-linked antibodies (Santa Cruz Biotechnology Inc.). The proteins were detected using the SuperSignal West Femto chemiluminescent substrate (Thermo Fisher Scientific, Rockford, IL, USA) and were then quantified using Quantity One Analysis Software (Bio-Rad).

### 2.7. Statistical Analysis

The statistical significance of the results was conducted by using one-way ANOVA, followed by Tukey's multiple tests. All reported data were expressed as the mean of triplicate measurements ± standard deviation (SD), and a *p* value lower than 0.05 was considered statistically significant.

## 3. Results

### 3.1. Physicochemical Properties of the Mixture of Extracts

The polyphenols from the mixture of extracts were identified by comparing the data from the chromatogram of the extract with a chromatogram of a standard solution. Compound detection was performed at several wavelengths: 235, 255, 259, 260, 270, 274, 280, 285, 310, 320, and 345 nm. The standard solution was prepared by mixing 1 mL of the stock standard solutions of synapic acid, myricetin, vanillic acid, quercetin, gallic acid, syringic acid, epicatechin, naringenin, p-coumaric acid, caffeic acid, and luteolin, and we injected in triplicate (Table 1).

The total and flavonoid polyphenol contents of the extract mixture are different depending on the ratio of the two associated extracts. The largest amount of total polyphenols from the analyzed plant extract combination was T : S (3 : 1), but it had a low content of total flavonoid (Table 2).

We observed that the extract combination T : S (1 : 2) had a well-balanced ratio regarding the bioactive compounds, i.e., 228.98 mg GAE/g DW content in total polyphenols and 8.05 mg QE/g dry weight (DW) total flavonoid. Thus, we evaluated the antioxidant activity of the plant extract combinations using the DPPH, ABTS, and FRAP methods. In Table3, the obtained results are summarized.

It was found that of the 5 combinations of plant extracts, in a different ratio of the two plant species studied, the combination that showed a better and balanced antioxidant activity is the combination of T : S (1 : 2) with ABTS of 19,175 *μ*mol of Trolox/mL and FRAP 40.994 *μ*mol Trolox/mL. The antioxidant activity measured by the DPPH method was 67.614%.

### 3.2. Antimicrobial Activity Evaluation

Table 4 shows the results obtained when testing the antimicrobial activity of the extracts.

There are several methods and techniques used for determining antimicrobial activity. Unfortunately, their sensitivity is not constant and comparable. Because of this, the results can be influenced by the chosen method. By assessing the inhibition zone diameter of the five plant extract combinations on the *Staphylococcus aureus* ATCC 25923 strain, it was observed that the antibacterial effect on the tested species was low to moderate. The most sensitive antimicrobial effects can be considered for the combination T : S 1 : 2 on the *Staphylococcus aureus.* For the *Streptococcus pneumonia* ATCC 49619, the inhibition zone diameter was moderate. Regarding the two wild strains isolated from human clinical cases, concerning the antibacterial effect of the T : S 1 : 2 combination, the most resistant strain was *Streptococcus agalactiae*, while *Streptococcus pyogenes* exhibited a weak one. An inhibition zone was also detected in the case of the *Staphylococcus aureus* ATCC 25923 strain and the *Streptococcus pneumonia* ATCC 49619 strain in the extract combination T : S 1 : 3, whereas in the case of the wild strains of *Streptococcus pyogenes*, the effect was reduced, and in *Streptococcus agalactiae*, there was no antibacterial effect. In the case of *Streptococcus agalactiae* isolated from human clinical cases, none of the extract combinations had an antimicrobial effect.

These results determined our choice for the combination of extracts that had the greater amount of flavonoids, better antimicrobial activity, and good viability test results in order to obtain a phytocomplex from the two extracts with anti-inflammatory properties used in atopic dermatitis.

### 3.3. Cell Viability

In order to test the efficacy of the combination of extracts in atopic dermatitis, fibroblasts exposed to bacterial lipopolysaccharides were used. The effects of natural compounds were evaluated by a viability test and inflammation marker assessment including IL-31, IL-33, IL-6, TNF-*α*, and STAT3 levels. In addition, the expressions of NF*κ*B and pNF*κ*B were quantified by western blot.

**Figure 1:**
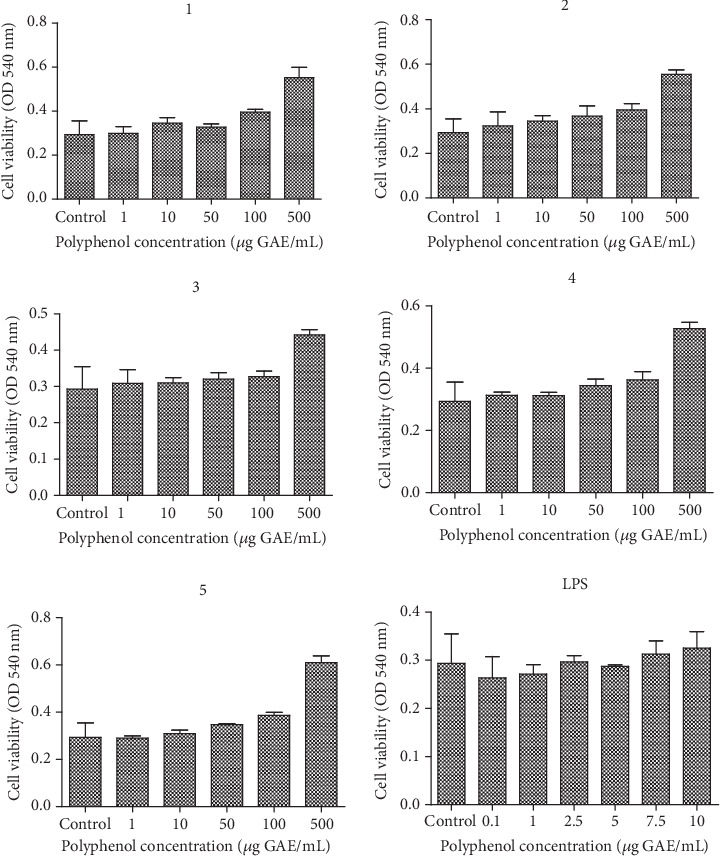
Cell viability of BJ cells treated with T : S extracts in different concentrations for 24 h, compared to untreated cells. BJ cells were exposed to T : S extracts in concentrations ranging between 0 and 500 *μ*g/mL compared to untreated cells; the two extracts were tested using different formulations, according to the following ratio between *Tropaeolum majus* L. (T) and *Salvia officinalis* L. extracts (S): (1) 1 : 1, (2) 1 : 2, (3) 2 : 1, (4) 3 : 1, and (5) 1 : 3. Data are presented as mean of OD540 ± SD, *n* = 3 for each sample.

The cell viability of BJ was evaluated with different formulations, according to the ratio between *Tropaeolum majus* L. (T) and *Salvia officinalis* L. extracts (S), respectively: (1) 1 : 1, (2) 1 : 2, (3) 2 : 1, (4) 3 : 1, and (5) 1 : 3. The results obtained showed an increase in viability at higher doses of polyphenols (50-500 *μ*g polyphenols/mL) in a dose-dependent manner, compared to untreated cells. When the BJ cells were exposed to different doses of LPS the viability was not affected (Figure 1).

**Figure 2:**
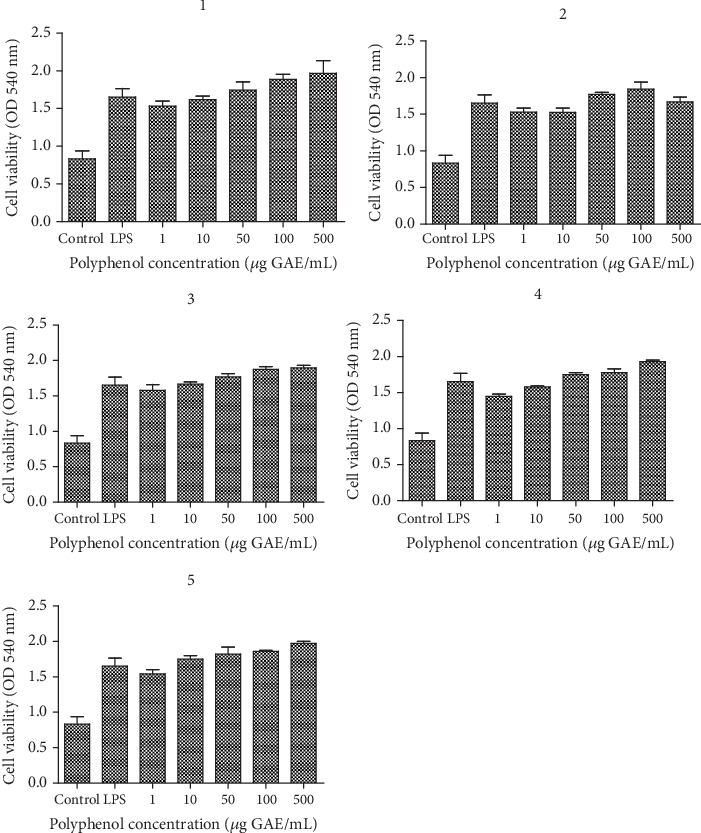
Cell viability of BJ cells treated with T : S extracts in different concentrations for 24 h, followed by exposure to LPS 10 *μ*g/mL compared to LPS and untreated cells. BJ cells were exposed to T : S extracts in concentrations ranging between 0 and 500 *μ*g/mL or only to LPS 10 *μ*g/mL compared to untreated cells; the two extracts were tested using different formulations, according to the following ratios between *Tropaeolum majus* L. (T) and *Salvia officinalis* L. extracts (S): (1) 1 : 1, (2) 1 : 2, (3) 2 : 1, (4) 3 : 1, and (5) 1 : 3. Data are presented as mean of OD540 ± SD, *n* = 3 for each sample.

BJ cells treated with polyphenols and LPS showed the same pattern of cell viability as cells exposed only to polyphenols or only to LPS (Figure 2). The highest cell viability was obtained at the highest concentration of polyphenols suggesting that the extracts used and LPS were not toxic. For the evaluation of inflammation under natural extracts and LPS, the 1 : 2 T : S formula was chosen.

### 3.4. Inflammation Marker Assessment

**Figure 3:**
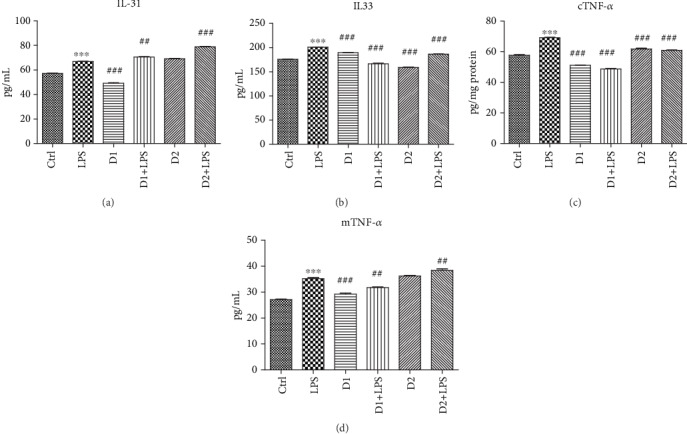
IL-31, IL-33, and TNF-*α* levels in supernates of BJ cells treated with T : S extracts in a 1 : 2 formula followed by exposure to LPS. Inflammatory markers in supernates of BJ cells exposed to T : S extracts in a 1 : 2 formula and LPS. Protein expressions of IL-31 (a), IL-33 (b), mTNF-*α* (c), and cTNF-*α* (d) (pg/mL) were determined by ELISA. The statistical significance between LPS-exposed groups and the control group or LPS-exposed groups and treated groups were evaluated with one-way ANOVA, followed by Tukey's posttest. Each bar represents mean ± standard deviation (*n* = 3); ^∗^*p* < 0.05, ^∗∗^*p* < 0.01, and ^∗∗∗^*p* < 0.001 vs. ctrl cells and ^#^*p* < 0.05, ^##^*p* < 0.01, and ^###^*p* < 0.001 vs. LPS.

The exposure of BJ cells to 10 *μ*g/mL LPS for 24 h induced IL-31 secretion (*p* < 0.001), while the administration of only high doses of the T : S extract in a 1 : 2 formula significantly reduced the IL-31 level compared to LPS (*p* < 0.001) (Figure 3). The administration of low doses of extract did not change IL-31 levels compared to LPS (*p* > 0.05). The association of both doses of extract with LPS administration maintained the high IL-31 values in BJ cells, suggesting the incomplete protective effect of the extract (*p* < 0.001 compared to LPS). The exposure of BJ cells to LPS significantly increased the IL-33 level (*p* < 0.001), while both doses of extracts administered in association with LPS diminished the IL-33 secretion (*p* < 0.001). The same pattern was observed when we measured the TNF-*α* levels in cell lysates after T : S associated with LPS administration. LPS exposure increased TNF-*α* secretion in cell lysates (*p* < 0.001), and high doses of extract alone or in combination with LPS reduced TNF-*α* levels (*p* > 0.001). In cell supernates, the inhibitory effect was obtained after high doses of extract (*p* < 0.001) or after high doses of extract administered in association with LPS (*p* < 0.001). The exposure to low doses of extract and LPS maintained TNF-*α* secretion at a low level (*p* < 0.001 compared to LPS) but it was significantly higher compared to those obtained after *D*_1_ + LPS (*p* < 0.001).

**Figure 4:**
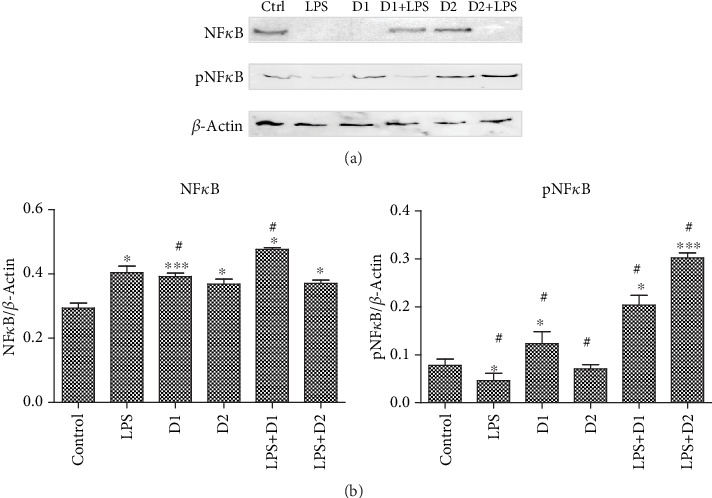
NF*κ*B and pNF*κ*B expressions in BJ cell lysates treated with T : S extracts in a 1 : 2 formula followed by exposure to LPS. Western blot analysis of NF*κ*B and pNF*κ*B expressions in BJ cells treated with T : S extracts in a 1 : 2 formula followed by exposure to LPS. Image analysis of WB bands was done by densitometry; results were normalized to *β*-actin. The statistical significance between LPS-exposed groups and the control group or LPS-exposed groups and treated groups were evaluated with one-way ANOVA, followed by Tukey's posttest. Each bar represents mean ± standard deviation (*n* = 3); ^∗^*p* < 0.05, ^∗∗^*p* < 0.01, and ^∗∗∗^*p* < 0.001 vs. ctrl cells and ^#^*p* < 0.05, ^##^*p* < 0.01, and ^###^*p* < 0.001 vs. LPS.

To evaluate the effect of the exposure to the T : S extract and LPS on NF*κ*B activation, we quantified the expression of constitutive and phosphorylated forms of NF*κ*B in BJ cell lysates by western blot (Figure 4). The addition of LPS (*p* < 0.05) or two doses of extract (*p* < 0.001 and *p* < 0.05) or LPS associated with extract (*p* < 0.05) to the culture medium led to an increase in the expression of NF*κ*B compared to untreated cells. The two doses of extract had a significant impact on NF*κ*B expression (*p* < 0.05) and reduced the value of NF*κ*B. The combination of LPS and high doses of extract significantly increased the NF*κ*B expression compared to LPS (*p* < 0.05), while the low dose (*D*_2_ + LPS) did not influence the NF*κ*B expression. The combined treatment (LPS and two doses of extract) increased the phosphorylated form of NF*κ*B. A similar effect was noticed only after high doses of extract (*p* < 0.05).

**Figure 5:**
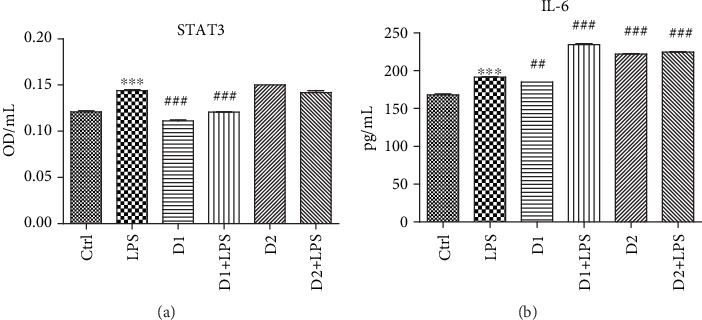
STAT3 and IL-6 levels in supernates of BJ cells treated with T : S extracts in a 1 : 2 formula followed by exposure to LPS. Proinflammatory marker (IL-6) and transcription factor (STAT3) in supernates of BJ cells exposed to T : S extracts in a 1 : 2 formula and LPS. Protein expressions of STAT3 (a) and IL-6 (b) were determined by ELISA. The statistical significance between LPS-exposed groups and the control group or LPS-exposed groups and treated groups were evaluated with one-way ANOVA, followed by Tukey's posttest. Each bar represents mean ± standard deviation (*n* = 3); ^∗^*p* < 0.05, ^∗∗^*p* < 0.01, and ^∗∗∗^*p* < 0.001 vs. ctrl cells and ^#^*p* < 0.05, ^##^*p* < 0.01, and ^###^*p* < 0.001 vs. LPS.

To evaluate the effect of LPS on BJ cells and the impact of the two doses of the T : S extract on the proinflammatory pathway modulated by NF*κ*B, we quantified the IL-6 levels and downstream signal transducer and activator of transcription (STAT3) in cell supernates (Figure 5). LPS exposure significantly increased the IL-6 and STAT3 levels (*p* < 0.001), while the pretreatment with high doses of extract maintained a low STAT3 level, comparable with the control. The same behaviour showed IL-6 secretion when only high doses of extract were used (*p* < 0.01). The association between both doses of extract and LPS exposure increased IL-6 secretion. A low dose of extract did not change the STAT3 expression when compared to cells exposed only to LPS (*p* > 0.05).

## 4. Discussion

Atopic dermatitis is one of the most common chronic inflammatory skin diseases especially in childhood and in industrialized countries, characterized by eczematous skin lesions and intense pruritus. The typical feature of the disease is the alteration of the skin barrier function with inflammation and cytokine type 2 production which leads to an increase of IgE level and amplifies the inflammation and skin barrier dysfunction. It had been shown that the colonization of skin lesions with *Staphylococcus aureus* or other bacterial, fungal, and viral agents reduced the commensal flora and aggravated the flares of the disease. It was demonstrated that *Staphylococcus aureus* produced the virulence factors such as biofilm, superantigens, *α*-toxin, and protein A which reduce the effectiveness of antibiotic treatment [47] and stimulate the immune system and are positively correlated with disease severity [48].

In the past years, new treatments have been developed, but sometimes these agents have side effects or are difficult to tolerate in the long term. Therefore, the use of natural agents such as licorice, green tea, soybeans, acai berries, turmeric, and pomegranate [49] became more frequent for the treatment of diseases and prevention of the relapses. *Salvia officinalis* L. and *Tropaeolum majus* L. were chosen as extracts for the experiment due to the following beneficial properties: antioxidant and protective capacity against side effects of different drugs; anti-inflammatory, antiseptic, and modulatory role in apoptosis; and antiproliferative ability. Thus, multiple applications of *Salvia officinalis* L. extract inhibited the skin lesions on NC/Nga mice and reduced the nerve growth factor- (NGF-) induced neuritic outgrowth in PC12 cells [50]. On an animal model of inflammation, *Salvia officinalis* L. reduced oxidative stress and inflammation induced by bacterial lipopolysaccharides and increased antioxidant enzyme activity [51]. Besides this, *Tropaeolum majus* L. extract suppressed the release of TNF-*α* and LTB_4_ from human peripheral blood mononuclear cells and downregulated extracellular signal-regulated kinase (ERK) 1/2 and c-Jun activation and diminished prostaglandin (PG) E_2_ synthesis induced by LPS [52].

Our study demonstrated that the T : S 1 : 2 formula and LPS were not toxic on normal fibroblasts and reduced the IL-33 and TNF-*α* levels, the last one both in cell lysates and supernates, and diminished the transcription factor STAT3. The low doses of extracts in combination with LPS did not reduce the IL-31 and mTNF-*α* secretions suggesting a partial protective effect of polyphenols and anthocyanins from extract composition on BJ cells, although only in high doses. The high doses of extracts in combination with LPS exposure significantly increased the protein NF*κ*B expression, while the low doses did not influence the constitutive NF*κ*B levels. Both doses of extracts enhanced the NF*κ*B activation but without the significant increase of protein synthesis. In addition, the *Tropaeolum majus* L. extract mixed with *Salviae folium* extract in a 1 : 2 formula exhibited a moderate antibacterial effect on *Staphylococcus aureus.*

Generally, fibroblasts are involved in the immune response of the skin in atopic dermatitis by active secretion of cytokines, chemokines, and growth factors [53]. In addition, these cells respond to cytokines secreted by other cells from the skin and are actively involved in remodelling and repair [54]. Therefore, we used dermal fibroblasts exposed to inflammatory stimuli to mimic the behaviour of cytokine secretion in atopic dermatitis.

The 1 : 2 formula was chosen due to *in vitro* biological properties. Thus, the results on the chemical composition of the five combinations of extracts of *Tropaeolum majus* L. and *Salvia officinalis* L. (Table 2) as well as data from the literature on the biological activity of polyphenols and flavonoids, led us to choose formula 2 for the evaluation of biological properties, namely the T : S ratio 1 : 2 (228.98 mg GAE/mL and 8.052 mg QE/mL in the fluid extract). This extract combination also had a good antioxidant activity demonstrated by different analytical methods used (DPPH, ABTS, and FRAP).

It is known that high amounts of reactive oxygen and nitrogen species (ROS) are produced in atopic dermatitis in correlation with the severity of inflammation. Therefore, the therapeutic use of antioxidant agents can reduce the inflammation and consequently the severity of disease. The combination proposed to be tested in our experiment demonstrated a good antioxidant activity *in vitro* with DPPH, ABTS, and FRAP methods.

It is known that IL-33 is an inflammatory cytokine that is overexpressed in the keratinocytes of patients with AD. The molecular mechanism involved suggested that IL-33 enhanced the maturation of the mast cells [55] and represented an important initiator of immune responses. Schmitz et al. noticed on an experimental animal model that the injection of IL-33 into mice induced an increase of eosinophilia [56] and production of superoxide anion and IL-8 [57]. IL-33 exerts its action via the ST2 receptor (a member of the toll-like/IL-1 receptor superfamily), also involved in the production of inflammatory factors such as IL-6, TNF-*α*, and leukotriene. IL-33, known as an “alarmin,” is stored in the cell nucleus, particularly in keratinocytes, and is released after cellular necrosis, in order to alert the innate immune system [58]. IL-33 is attached to specific complex receptor ST2 (IL-1RL1) and the IL-1 receptor (IL-1RAcP) helper protein serving as coreceptor to initiate the immune cascade and produce IL-5, IL-13, and IL-4. Activation of ST2 and ILC2s in keratinocytes and overexpression of IL-33 can lead to the development of atopic dermatitis [59]. IL-33 can also increase LPS-induced production of TNF-*α*, IL-6, and IL-1*β* from mouse macrophages suggesting the promotion of proinflammatory processes [60]. Sato et al. [61] found an increase of IL-33 levels in macrophages exposed to LPS. Therefore, the inhibition of IL-33 production may be a good target for the suppression of inflammation in AD.

In our study, the mixture of two extracts reduced the IL-33 secretion induced by LPS, particularly in high doses. The same pattern was observed on TNF-*α* levels. LPS increased TNF-*α* secretion in BJ cells, but the effect diminished by a high dose of extract in medium and both doses of extract in cell lysates. The protective effect of the tested formula on IL-31 was important only on cells without LPS exposure, especially in a high dose; in the presence of LPS, the IL-31 levels increased mainly at a low dose of the extract suggesting the partial efficiency of the protective effect. It is known that IL-31 belongs to the IL-6 cytokine family, and it is produced in leukocytes after IL-33 stimulation, and together they can stimulate chemokines on AD [62]. IL-33 in turn downregulated the expression of claudin-1 in keratinocytes through the STAT3 pathway [63]. Moreover, after binding to its receptor, IL-31 can induce the phosphorylation of JAK1/2 and PI3K-Akt and trigger the activation of the STAT pathway especially STAT3 and STAT5 [64]. It is known that both IL-31 and IL-33 initiate skin inflammation and lead to the dysregulation of immunomodulatory proteins in atopic dermatitis through STAT3 pathway activation. In our study, this effect was diminished by pretreatment with high doses of extract demonstrating the anti-inflammatory activity of the mixture of extracts.

STAT3 is a transcription factor that becomes activated by phosphorylation in response to ligands such as IL-6, IL-5, interferons, or growth factors [65]. Our results showed that STAT3 and IL-6 levels increased in parallel in cells exposed to LPS and diminished when the cells were pretreated with high doses of extract or high doses and LPS, especially STAT3. The low doses of extract increased IL-6 significantly while STAT3 was not influenced. It is known that the proinflammatory effect of IL-6 requires the activation of the canonical pathway IL-6/JAK/STAT3, which phosphorylates STAT3 and induces its translocation to the nucleus. STAT3 is a transcription factor which mediates the expression of genes involved in inflammation, cell growth, differentiation, and survival or apoptosis [66]. Moreover, STAT3 is necessary for the differentiation of Th17 helper T cells, known as cells involved in a variety of autoimmune diseases including atopic dermatitis [67]. Even though the new formula of extracts stimulated the inflammation partially, in high doses, T : S 1 : 2 inhibited the transcription of different key factors involved in the activation of eosinophils, in the maturation of B cells, and the suppression of regulatory T cells, or in the production of proinflammatory cytokines and proangiogenic molecules.

The cytokines involved in the pathogenesis of atopic dermatitis are regulated by some other transcription factors including NF*κ*B and AP-1. The NF*κ*B family of transcription factors regulates the expression of a broad range of genes involved in inflammation, development, cell proliferation, survival, differentiation, and senescence [68]. NF*κ*B is located in the cytosol in an inactive state by a family of inhibitory proteins, including I*κ*B family members and related proteins. The best studied protein of this family is I*κ*B*α*. After phosphorylation of the I*κ*B*α* protein and its degradation, NF*κ*B is translocated into the nucleus where it induces expression of multiple genes involved in inflammatory response [69–71]. The activation of NF*κ*B by the classical pathway involves various stimuli, such as ligands of cytokine receptors, pattern-recognition receptors (PRRs), TNF receptor (TNFR) superfamily members, as well as T-cell (TCR) and B-cell receptors [72]. NF*κ*B promotes the transcription of Th2 cytokines, such as IL-6 and ICAM-1 [73], which in turn activates NF*κ*B.

Generally, the bioactive compounds with an antioxidant activity modulate the expression of transcription factors such as AP-1 and NF*κ*B and control the proinflammatory enzyme activities of phospholipase A2, cyclooxygenase, lipoxygenase, and nitric oxide synthase [74–76]. In our study, LPS exposure is associated with a high expression of NF*κ*B, while the two doses of extract reduced the NF*κ*B levels demonstrating the beneficial role of the extract. The constitutive form of NF*κ*B was not influenced by low doses of extract administered before LPS exposure while the association of LPS and high doses of extract increased the NF*κ*B constitutive form and its activation. A similar effect on the NF*κ*B activation was induced by exposure to LPS and low doses of extract, suggesting a major influence of the extract on NF*κ*B phosphorylation and less on protein secretion.

## 5. Conclusion

In conclusion, in the present study, we bring important knowledge regarding the interaction of BJ cells, LPS, and two doses of a mixture of extracts in order to simulate the pathogenetic mechanisms involved in AD. The results demonstrated that the mixture of two extracts in an optimum formula exhibited antibacterial activity on *Staphylococcus aureus* and inhibited the secretion of inflammatory cytokines, especially IL-33 and TNF-*α* in dermal fibroblasts exposed to LPS and diminished the secretion of transcription factor STAT3 involved in the perpetuation of inflammation and aggravation of skin lesions. The evidence provided above indicates that the mixture of two extracts in a 1 : 2 formula is a potential therapeutic candidate for conditions associated with inflammation including atopic dermatitis. However, further studies with multiple doses and exposure times are necessary to demonstrate the real clinical efficiency in atopic dermatitis.

## Figures and Tables

**Table 1:** The composition of the two extracts in RP-HPLC analysis.

Bioactive compounds	*λ*_max_	Plant extract (mg/kg)
Epicatechin	280	2430.99 ± 1.05
Luteolin	345	4379.88 ± 1.35
Naringenin	285	1098.44 ± 2.5.5
Quercetin	260	425.04 ± 0.65
Myricetin	255	44.02 ± 0.08
Synapic acid	235	101.61 ± 1.23
P-coumaric acid	310	757.21 ± 7.08
Caffeic acid	320	1552.14 ± 1.25
Galic acid	270	1581.79 ± 1.03
Vanillic acid	259	283.27 ± 5.09
Syringic acid	274	895.25 ± 1.22

Results are expressed as mean ± standard deviation.

**Table 2:** The content of polyphenols and flavonoids in the mixture of extracts.

Total in bioactive compounds	T : S (1 : 1)	T : S (1 : 2)	T : S (2 : 1)	T : S (3 : 1)	T : S (1 : 3)
Content in total polyphenols (mg GAE^∗^/g DW)	309.91	228.98	390.84	431.30	188.52
Total flavonoids (mg QE^∗∗^/g DW)	6.43	8.05	4.82	4.01	8.86

^∗^GAE: gallic acid; ^∗∗^QE: quercetin.

**Table 3:** Antioxidant activity determined by the three chemical methods of the samples.

Sample	DPPH%	ABTS (*μ*mol Trolox equivalent/mL)	FRAP (*μ*mol Trolox equivalent/mL)
T : S (1 : 1)	71.836	19.826	44,919
T : S (1 : 2)	67.614	19.175	40.994
T : S (2 : 1)	76.057	20.478	48.842
T : S (3 : 1)	78.168	20.804	50.704
T : S (1 : 3)	65.505	28.851	39.034

**Table 4:** Antimicrobial activity of the five plant extract combinations.

Strain	*Tropaeolum maju*s	*Salvia officinalis*	T : S 1 : 1	T : S 1 : 2	T : S 1 : 3	T : S 2 : 1	T : S 3 : 1	PEN 10 U	Distilled water
*S. aureus* ATCC 25923	6	13	17	20	18	16	16	37	0
	6	12	16	20	18	16	12	38	
	6	12	15	19	17	15	13	38	

Mean	6	12.33	16	19.66	17.66	15.66	13.66	37.66	

*S. pneumoniae* ATCC 49619	8	9	13	14	18	17	18	27	0
	8	9	13	15	17	18	20	27	
	9	8	14	14	19	19	16	27	

Mean	8.33	8.66	13.66	14.66	18	18	18	27	

*S. pyogenes* from human clinical cases	8	9	10	10	12	10	21	41	0
	8	10	9	9	14	11	21	40	
	9	11	11	12	13	11	18	41	

Mean	8.33	10	10	10.33	13	10.66	20	40.66	

*S. agalactiae* from human clinical cases	6	8	0	0	0	0	0	35	0
	6	8						35	
	6	8						34	

Mean	6	8						34.66	

## Data Availability

The data used to support the findings of this study are included within the article.
